# Randomized Evaluation of Beta Blocker and ACE-Inhibitor/Angiotensin Receptor Blocker Treatment for Post Infarct Angina in Patients With Myocardial Infarction With Non-obstructive Coronary Arteries: A MINOCA-BAT Sub Study Rationale and Design

**DOI:** 10.3389/fcvm.2021.717526

**Published:** 2021-10-08

**Authors:** Sivabaskari Pasupathy, Bertil Lindahl, Rosanna Tavella, Anna M. Nordenskjöld, Christopher Zeitz, Margaret Arstall, Matthew Worthley, Christopher Neil, Kuljit Singh, Stuart Turner, Adil Rajwani, John Mooney, John F. Beltrame

**Affiliations:** ^1^Faculty of Health Sciences, The University of Adelaide, Adelaide, SA, Australia; ^2^Department of Cardiology, Central Adelaide Local Health Network, Adelaide, SA, Australia; ^3^Basil Hetzel Institute, Adelaide, SA, Australia; ^4^Department of Medical Sciences and Uppsala Clinical Research Center, Uppsala University, Uppsala, Sweden; ^5^Department of Cardiology, School of Medical Sciences, Örebro University, Örebro, Sweden; ^6^Department of Cardiology, Northern Adelaide Local Health Network, Adelaide, SA, Australia; ^7^Department of Medicine, University of Melbourne, Melbourne, VIC, Australia; ^8^Department of Medicine, Griffith University, Gold Coast, QLD, Australia; ^9^Department of Cardiology, John Hunter Hospital, Newcastle, NSW, Australia; ^10^Department of Cardiology, Royal Perth Hospital, Perth, WA, Australia; ^11^Department of Cardiology, Gosford Hospital, Gosford, NSW, Australia

**Keywords:** myocardial infarction, coronary angiogram, normal coronary angiography, non-obstructive coronary artery disease (NOCAD), Myocardial Infarction with Non Obstrucrive Coronary Arteries (MINOCA)

## Abstract

**Introduction:** Myocardial infarction with non-obstructive coronary arteries (MINOCA) occurs in ~10% of all patients with acute myocardial infarction (AMI), with an over-representation amongst women. Remarkably, it is estimated that as many as 1 in 4 patients with MINOCA experience ongoing angina at 12 months despite having no flow-restricting stenoses in their epicardial arteries. This manuscript presents the rationale behind Randomized Evaluation of Beta Blocker and Angiotensin-converting enzyme inhibitors/Angiotensin Receptor Blocker Treatment (ACEI/ARB) for Post Infarct Angina in MINOCA patients—The MINOCA BAT post infarct angina sub study.

**Methods:** This trial is a registry-based, randomized, parallel, open-label, multicenter trial with 2 × 2 factorial design. The primary aim is to determine whether oral beta blockade compared with no oral beta blockade, and ACEI/ARB compared with no ACEI/ARB, reduce post infarct angina in patients discharged after MINOCA without clinical signs of heart failure and with left ventricular ejection fraction ≥40%. A total of 664 patients will be randomized into four groups; (i) ACEI/ARB with beta blocker, (ii) beta blocker only, (iii) ACEI/ARB only, or (iv) neither ACEI/ARB nor beta blocker and followed for 12 months.

**Results:** The trial is currently recruiting in Australia and Sweden. Fifty six patients have been recruited thus far. Both sexes were equally distributed (52% women and 48% men) and the mean age was 56.3 ± 9.9 years.

**Conclusions:** It remains unclear whether conventional secondary preventive therapies are beneficial to MINOCA patients in regard to post infarct angina. Existing registry-based literature suggest cardioprotective agents are less likely to be used in MINOCA patients. Thus, results from this trial will provide insights for future treatment strategies and guidelines specific to MINOCA patients.

## Introduction

Myocardial Infarction with Non-Obstructive Coronary Arteries (MINOCA) refers to patients presenting with acute myocardial infarction (AMI) without angiographically evident obstructive (defined as ≥50% diameter stenosis) coronary artery disease (CAD). The reported prevalence of MINOCA has been between 5 and 14% with most recent large AMI registries reports ~10% of AMI population identified as MINOCA (1). Compared to conventional AMI patients (AMI with obstructive CAD–MICAD), MINOCA patients are more likely be females, younger and have fewer cardiovascular risk factors (2). The underlying pathophysiological mechanisms are poorly understood in these patients, although several different ischemic mechanisms have been proposed including plaque disruption, coronary artery spasm, microvascular dysfunction, thromboembolism, and coronary dissection (3). Furthermore, short- and long-term prognosis of MINOCA patients is comparatively more favorable than those with MICAD and worse as compared to the general population (4, 5). Moreover, Grodzinsky et al. (6) compared the post-MI angina and health status during the MI hospitalization and at 1, 6, and 12-months following MI using the Seattle Angina Questionnaire (SAQ) between MINOCA and those with MICAD. MINOCA patients had a high prevalence of angina during follow-up, with one in four patients reporting angina at 1 year after MI. In addition, MINOCA patients also reported worse disease-specific and generic health status at 12 months post-MI, including worse quality of life due to angina and lower satisfaction with the treatment of their angina (6).

Regarding medical management of these patients, clinical registry based studies suggest MINOCA patients are often less likely to receive secondary preventive medications when compared to MICAD patients (7–9). However, studies have reported benefits of ACEI/ARB therapy (10–13), statin therapy (11, 14), and beta blocker (15) therapy in MINOCA. In particular, Lindahl et al. (13) using data from the Swedish Web-system for Enhancement and Development of Evidence-based care in Heart disease Evaluated According to Recommended Therapies (SWEDEHEART) registry of 9,136 MINOCA patients showed that 24% experienced a Major Adverse Coronary Events (MACE) during a mean follow-up period of 4.1 years. The MACE components included a mortality of 13.4%, non-fatal MI in 7.1%, non-fatal stroke in 4.3%, and heart failure hospitalization in 6.4%. In propensity score matched comparisons, there was a significant 18% reduction in experiencing a MACE with Angiotensin-converting enzyme inhibitors/Angiotensin Receptor Blocker Therapy (ACEI/ARB) and a non-significant 14% trend with the use of beta-blockers, compared with respective control groups (13). This observational data is being evaluated in an international multicenter, randomized, controlled, open-label clinical study assessing the impact of ACEI/ARB and/or beta blockers on MACE, with the study methods recently published (16). This manuscript details the methods used in the ***MINOCA BAT Post Infarct Angina sub-study***, which is embedded within the MINOCA BAT Trial utilizing its infrastructure to investigate the post-infarct anti-anginal benefit of Beta Blocker and ACEI/ARB in MINOCA patients.

## Methods

MINOCA-BAT Post-infarct angina trial is a prospectively designed sub-study of the MINOCA-BAT trial; an investigator-initiated, registry-based, pragmatically designed, international, multicenter, open-labeled, 2 × 2 factorial design, randomized controlled trial (RCT) evaluating the impact of beta-blockers and/or ACEI/ARB on MACE. In the Post-infarct angina substudy, the impact of these therapies on health outcomes will be evaluated. Recruitment has commenced in Australia and Sweden with funding obtained from local sources, including the not-for-profit The Hospital Research Foundation in Australia and the Swedish Government's Swedish Research Council in Sweden. The intention to treat principle will be applied to analyze the study outcomes. The study is currently recruiting in Australia and Sweden.

The primary aim of this study is to determine whether oral beta-blocker and/or ACEI/ARB in patients with MINOCA and LV-EF ≥ 40% without clinical signs of heart failure impacts on post-infarct angina at 12 months, where angina frequency is assessed with SAQ-7. The secondary aims are to determine the impact of these treatment in (i) angina frequency, physical limitation, quality of life and patient satisfaction as scored by the SAQ-7 at 12 months. (ii) health related quality of life by EQ 5D at 12 months and (iii) mental health and depression scored by PHQ at 12 months.

### Definition of MINOCA

The definition of MINOCA has evolved over the years. In this study, utilizing the pragmatic approach, MINOCA is defined as per the European Society of Cardiology working group criteria (15). As per contemporary AMI guidelines (17), the MINOCA patients should have evidence of myocardial ischemia as the underlying cause for the myocardial injury. Therefore, in this study, efforts are made to exclude non-ischemic and non-cardiac conditions that could mimic the presentation of acute MI such as Takotsubo, Myocarditis, pulmonary embolism, etc.

### Hypothesis and Study End Points

The hypothesis of this study is treatment with beta blockers and/or ACEI/ARBs will reduce angina frequency in MINOCA patients with preserved LV-EF. The primary endpoint is to assess the angina frequency measured by SAQ-7 at 12 months. Secondary endpoints are health status measurement scores at 12 months as follows: (a) Angina stability, physical limitation, quality of life and patient satisfaction as scored by the SAQ-7. (b) Health related quality of life by EQ 5D. (c) Mental health and depression scored by PHQ 9. The specifics of these instruments are detailed in Table 1. Safety endpoints include readmission because for second- or third-degree AV-block, ventricular tachycardia, ventricular fibrillation, hypotension, acute kidney injury, syncope, or need for pacemaker.

**Table 1:** Parameters and validity of health-related quality of life instruments.

**Questionnaire**	**Parameters assessed**	**Validation References**
SAQ-7	A disease specific questionnaire with 7 questions that quantifies the impact of angina on health status of patients. •Physical Limitation (3 items) •Angina Frequency (2 items) •Quality of Life (2 items)	(18)
EQ-5D	General health related questionnaire with 5 questions assessing mobility, self-care, usual activities, discomfort, and depression of patients.	(19)
PhQ 9	Multipurpose questionnaire with 9 questions which assist in diagnosing, monitoring, and measuring the severity of depression in patients.	(20)

*SAQ, Seattle Angina Questionnaire; EQ-5D, EuroQol- 5Dimension; PhQ, Patient Health Questionnaire*.

### Inclusion/Exclusion Criteria

Patients above 18 years of age with a clinical diagnosis of MINOCA as shown in Table 2 with LV-EF ≥40% (assessed by left ventriculography, echocardiography or cardiac magnetic resonance imaging) will be approached to participate in this trial. Patients will be excluded if they have any of the following conditions that may affect their ability to comply with study protocol: pregnancy, prior revascularization treatment (e.g., percutaneous coronary intervention), diagnosis of myocarditis, takotsubo or heart failure, and any known contraindication or new indication or continuation to the study drugs.

**Table 2:** Definition of MINOCA as described in the MINOCA-BAT Trial (16).

1)	AMI-as per the Universal MI Criteria (17) •Positive cardiac biomarker (preferably troponin) defined as a rise and/or fall in serial levels, with at least one value above the 99th percentile upper reference limit and •Corroborative clinical evidence of infarction evidenced by at least one of the following:
	° Symptoms of ischemia
	° New/presumed new significant ST-T changes or left bundle branch block
	° Development of pathological Q waves
	° Imaging evidence of new loss of viable myocardium or new regional wall motion abnormality
	° Intracoronary thrombus evident on angiography or at autopsy
2)	Non-obstructive coronary arteries on angiography-defined as the absence of obstructive CAD on angiography (i.e., no coronary artery stenosis ≥50%), in any major epicardial artery. This include both patients with: •Normal coronary arteries (no stenosis >30%) •Mild coronary atheromatosis (stenosis >30% but <50%)^*^.
3)	No clinically overt-specific cause for the acute presentation: At the time of angiography, the underlying cause of the clinical presentation and myocardial injury is not apparent (e.g., clinically strong suspicion of myocarditis, Takotsubo syndrome^†^, or pulmonary embolism)

* *In borderline cases where it is difficult to determine visually whether a stenosis is below the 50% cut-off, a fractional flow reserve (FFR) or instaneous wavefress ratio (IFR) may be performed. FFR > 0.80 or IFR > 0.90 in the stenosis is considered to be non-significant and the patient is possible to include*.

† *Patients showing (transient) regional wall motion abnormalities (hypokinesia, akinesia, or dyskinesia) in the left ventricule extending beyond a single epicardial vascular distribution at the initial left ventriculogram or echocardiographic examination are considered to have a strong suspicion of Takotsubo syndrome and should not be included in the MINOCA-BAT trial. If these findings are not present the suspicion of Takotsubo syndrome is not considered strong and the patient can be included in the trial*.

### Study Protocol, Randomization, Participating Countries, and Ethics

**Figure 1:**
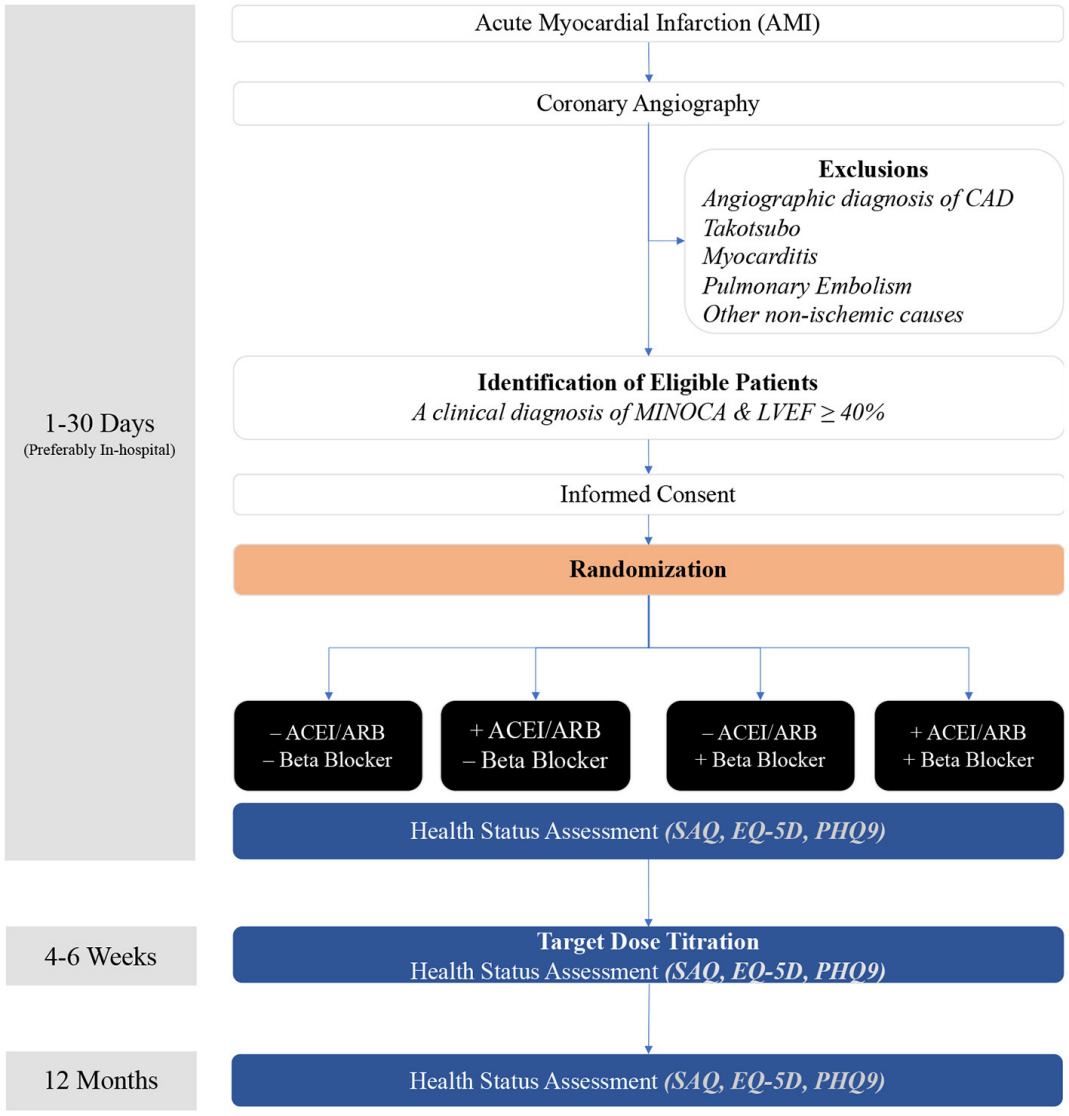
Study flow chart.

This registry based RCT will operate within the established Coronary Angiogram Database of South Australia (CADOSA) and SWEDEHEART (Sweden) Registries, where patients with MINOCA will be readily identified following coronary angiography. Additional sites without the registries will identify patients via their treating cardiology departments. Efforts are encouraged to exclude non-ischemic conditions such as clinically undetected myocarditis prior to randomization. Patients fulfilling the inclusion criteria and without any exclusion criterion will, after signing the informed consent form, be randomized on day 1–30 after index MINOCA. The data collection is performed by trained clinical trial staff. The study timeline overview is shown in Figure 1. The study is approved by Central Adelaide Local Health Network Human Research Ethics Committee (HREC/18/CALHN/157) in Australia and by relevant ethics review board in Sweden. ANZCTR Registration: ACTRN12618001858280.

The randomization will be performed in the module using permuted block randomization with 1:1:1:1 ratio, stratified by country. Randomization can be performed at any time day 1–30 after index MI. Patients may be randomized the same day as index AMI, e.g., day 1. The patients will be randomized to either beta blocker alone, ACEI/ARB alone or both beta blocker and ACE/ARB, or neither beta blocker nor ACEI/ARB. If randomized to study drug groups, the investigator will record the agent(s) chosen, the starting dose(s) and target dose(s) planned in the case report form. Control group patients randomized to neither beta blocker nor ACEI/ARB will not receive these study drugs.

Consistent with the pragmatic design and absence of industry support, the study drug class will be specified by the randomization protocol but not the specific agent, which will be determined by local practices. In Australia, the preferred study treatments will be atenolol and perindopril/candesartan, whereas in Sweden it will include bisoprolol/metoprolol and ramipril/enalapril/losartan/candesartan; however, the choice will be at the discretion of the treating clinician. The regional preferred treatments, including initial and target doses are summarized in Table 3. At the 6-week follow-up visit, the current dosage of the study treatments will be noted. This visit will be achieved via phone/on-line follow-up. Other therapies will be at the treating clinician's discretion but will be recorded at the follow-up visits.

**Table 3:** Preferred study treatments in Australia & Sweden.

	**Agent**	**Dose**
Beta blocker	Atenolol (Australia)	25–50 mg daily
	Bisoprolol (Sweden)	5–10 mg daily
	Metoprolol succinate (Sweden)	100–200 mg daily
ACEI	Perindopril Erbumine (Australia)	2–8 mg daily
	Perindopril Arginine (Australia)	2.5–10 mg daily
	Ramipril (Sweden)	5–10 mg daily
	Enalapril (Sweden)	10–20 mg daily
ARB	Candesartan (Australia)	4–16 mg daily
	Candesartan (Sweden)	16–32 mg daily
	Losartan (Sweden)	50–100 mg daily

Patients will be followed-up by a visit at 4–6 weeks after randomization and then at 12 months. The study commenced with patients enrolled in Australia in January 2019 and in Sweden May 2021. The adherence to the randomized drug(s) will be assessed at the follow-up visits at 4–6 weeks, and 12 months after randomization.

### Outcomes

Randomized patients will be followed regarding angina status, quality of life, mental health status, atrial fibrillation, renal failure, second- or third-degrees AV-block, hypotension, syncope and pacemaker implantation. These will be assessed at baseline, 4–6 weeks and 12 months, with the primary endpoint be the 12-month angina frequency.

### Safety

The safety data will be extracted directly from patients during follow up and from hospital/medical records or administrative data at the end of follow up. In addition, participants will be queried on any hospital admissions during the follow ups. The study steering committee appointed a data safety monitoring board (DSMB), comprised of three senior clinicians, without any affiliation with the Sponsor, the Coordinating Investigator, or local Investigators and with previous experience of working in a DSMB. The DSMB will oversee the safety of the study drugs and the general execution of the trial on behalf of the trial participants.

### Data Analysis

The data will be inspected graphically to establish normality prior to analysis. To identify potential confounders, chi squared or Student's *t*-test will be performed, to test the association between demographic or clinical characteristics of the sample at baseline and Angina Frequency as measured by the SAQ. For all endpoints, differences at baseline and 12 months between the beta-blocker treatment groups and the ACEI/ARB treatment groups will be analyzed using a repeated measures ANCOVA, adjusting for potential confounders. In addition, to investigate for trends over time in the SAQ Angina Frequency, score between the treatment groups, using all 4-time points in a mixed model approach. Subjects with missing data will be included in analyses, as data collection at every time point does not need to be adhered to in mixed models. Similar analyses will also be performed for the EQ-5D and PHQ-9.

### Sample Size

The study by Grodzinsky et al. (6) reported a post-infarct SAQ angina frequency score of 85 ± 21, thus, to detect a 20% change in SAQ angina frequency with a power of 0.9 at the alpha 0.05 level, 149 patients/study group are required (i.e., 596 MINOCA patients). Allowing for a 10% dropout then 664 patients will be recruited into this trial. The data collection will continue until the target number is achieved.

## Results

By 12 May 2021, 56 patients have been recruited in the study, 54 in Australia and 2 in Sweden respectively. Men and women were equally distributed (52% women and 48% men) and the mean age was 56.3 ± 9.9 years.

## Discussion

MINOCA is now an established clinical entity with heterogenous pathophysiology. Despite being apparent as early as the 80s, the management of these patients has not been addressed. It cannot be assumed that proven therapies in AMI patients with obstructive CAD are effective in MINOCA since (a) AMI pathogenetic mechanisms differ, (b) the prognosis differs with reduced mortality and re-infarction, (c) observational data suggest that some established therapies in MICAD (i.e., dual anti-platelet agents) are ineffective in MINOCA. Accordingly, randomized controlled clinical trials are required to identify the optimal therapy for patients with MINOCA.

### Minoca Bat Trial and Post Infarct Angina Sub Study

The ongoing main MINOCA BAT trial (16) will address the impact of ACEIs/ARBs and beta-blockers on MACE (defined as all-cause mortality, readmission because of MI, ischemic stroke, or heart failure) by randomizing ~3,500 patients with MINOCA. The post-infarct angina sub study will provide insights into the effectiveness of the trial therapies in controlling post-infarct angina, nestled within the construct of the main MINOCA-BAT clinical trial. Since the ischemic pathophysiology in MINOCA may differ to MICAD [i.e., coronary vasospasm, coronary microvascular dysfunction, coronary embolism/thrombosis, or spontaneous coronary artery dissection (21) may contribute], then conventional therapies may be ineffective.

Beta-blockers are a well-established anti-anginal agents in patients with obstructive CAD (22) and are effective in patients with coronary microvascular dysfunction (23); however they may be detrimental in patients with epicardial coronary artery spasm (24). Thus, whether their use will prevent/limit the occurrence of post-infarct angina in the heterogenous MINOCA disorder will be a key finding of this study. Furthermore, ACEI have been shown to have anti-anginal effects in patients with coronary microvascular dysfunction (25), although their benefits in other coronary vasomotor disorders remains unclear. Hence this study will provide key insights into the impact of blocking beta receptors and inhibiting the renin-angiotensin pathway.

## Strength and Limitations

The strength of this study is its pragmatic design as it evaluates effects of treatment in a real-world context; a positive result in this study can inform practice because it provides evidence that the treatment/intervention is effective in routine clinical practice. The findings of the study will be limited by (i) the undefined heterogeneity in pathophysiologic ischemic mechanisms—i.e., unable to delineate which patients have coronary spasm, microvascular dysfunction, or other causes of the myocardial infarct; (ii) therapeutic heterogeneity—i.e., variability in specific agents and dosages used within the randomized treatment drug classes; (iii) pharmacokinetic heterogeneity of drugs used within the class (iv) open label study design; (v) patients without a predisposition for ongoing angina may be randomized. The impact of some of these prospectively identified limitations will become apparent on the completion of the trial.

## Conclusions

MINOCA patients are less likely to receive cardio-protective therapies and have a significant prevalence of post infarct angina and poor quality of life. There are no clinical trials to date that have examined the benefit of these agents in MINOCA and thus there are no clinical guidelines currently available for the preventive management of these patients. There is a clear need to study these patients post infarct angina management strategies and this study is designed to meet this need.

## Data Availability Statement

The raw data supporting the conclusions of this article will be made available by the authors, without undue reservation.

## Ethics Statement

The studies involving human participants were reviewed and approved by Central Adelaide Local Health Network Human Research Ethics Committee. The patients/participants provided their written informed consent to participate in this study.

## Author Contributions

SP wrote the first draft of the manuscript. JB and BL critically revised the manuscript. All authors contributed to manuscript revision, read, and approved the submitted version.

## Funding

The Australian study sites are supported by the funding from The Hospital Research Foundation, a not-for-profit organization, which provides research grants to South Australian public hospitals. The grant awarded was entitled The Basil Hetzel Translational Grant 2017. The Swedish Government's Swedish Research Council have contributed to the Swedish part of the study as well as for design and maintenance of the database and the international randomization module (Grant 2017-00478).

## Conflict of Interest

The authors declare that the research was conducted in the absence of any commercial or financial relationships that could be construed as a potential conflict of interest.

## Publisher's Note

All claims expressed in this article are solely those of the authors and do not necessarily represent those of their affiliated organizations, or those of the publisher, the editors and the reviewers. Any product that may be evaluated in this article, or claim that may be made by its manufacturer, is not guaranteed or endorsed by the publisher.
